# Poster Session I - A102 GENDER-SPECIFIC BARRIERS IMPACTING THE GASTROENTEROLOGY TRAINING JOURNEY: A SCOPING REVIEW

**DOI:** 10.1093/jcag/gwaf042.102

**Published:** 2026-02-13

**Authors:** H R Tran, V W Lo, Y Song, M Fields, C M Walsh

**Affiliations:** McMaster University, Hamilton, ON, Canada; McMaster University, Hamilton, ON, Canada; McMaster University, Hamilton, ON, Canada; McMaster University, Hamilton, ON, Canada; Paediatrics, The Hospital for Sick Children, Toronto, ON, Canada

## Abstract

**Background:**

Women remain underrepresented among trainees and attendings in Gastroenterology (GI), in Canada and globally. Although gender-specific challenges for women in GI have been identified, the barriers influencing residents’ and fellows’ career decision-making related to GI have not been systematically reviewed.

**Aims:**

To explore gender-specific factors influencing Internal Medicine and Pediatrics residents’ decisions to pursue GI training, and GI fellows’ decisions to pursue subspecialty fellowship training.

**Methods:**

A scoping review was conducted following the PRISMA-ScR checklist and Arksey and O’Malley framework. Published studies from January 1 1974 to April 25 2025 were identified from Ovid MEDLINE and Embase databases. Reference lists of included articles were also manually searched. Eligible studies included English-language abstracts or manuscripts identifying barriers influencing the decision to pursue GI training among Internal Medicine and Pediatrics residents, or advanced training among GI fellows. Two independent reviewers screened titles, abstracts and full-texts using Covidence, with data extracted in duplicate on study design, participant characteristics, barriers examined, and key findings. Data were coded using 8 themes from the American Gastroenterology Association (AGA) Gender Equity Framework.

**Results:**

A total of 2731 studies were identified, with 20 included for data extraction. Most were cross-sectional (95%), published after 2020 (75%), from the United States (60%), and focused on trainees in adult programs (85%). Commonly identified barriers mapped to themes of family planning and responsibilities (80%), mentorship (70%), and gender-based bias (50%). Less common barriers related to learning environment (40%), wellbeing (35%), leadership and career advancement (30%), and agency (25%). New themes generated included gender representation in the workplace, length of training, and perceived competitiveness. Mentorship was a key factor, with same-gender mentorship seen as positive and the absence of mentorship as a deterrent. Family planning was important; maternity leave policies were viewed as supportive, but challenges with childcare responsibilities was a barrier. Perceived negative interactions with staff and peers, gender-based pay disparities, and microaggressions were identified as deterrents to pursuing additional GI-focused training. Well-being issues, including high burnout risk, deter residents from pursuing training, while fellows emphasized challenges with work-life balance.

**Conclusions:**

There are common gender-specific challenges to advancing along the GI training pathway among trainees, specifically family planning and mentorship. The AGA Gender Equity Framework effectively characterized these gender-specific barriers. Future studies should explore trainee experiences in Pediatrics and non-Western settings.

**Funding Agencies:**

None

